# X-ray Dark-Field Imaging for Improved Contrast in Historical Handwritten Literature

**DOI:** 10.3390/jimaging8090226

**Published:** 2022-08-24

**Authors:** Bernhard Akstaller, Stephan Schreiner, Lisa Dietrich, Constantin Rauch, Max Schuster, Veronika Ludwig, Christina Hofmann-Randall, Thilo Michel, Gisela Anton, Stefan Funk

**Affiliations:** 1 Erlangen Centre for Astroparticle Physics (ECAP), Friedrich-Alexander-Universität Erlangen-Nürnberg, Erwin-Rommel-Str. 1, 91058 Erlangen, Germany; 2 Universitätsbibliothek Handschriften und Graphische Sammlung, Friedrich-Alexander-Universität Erlangen-Nürnberg, Universitätsstraße 4, 91054 Erlangen, Germany

**Keywords:** X-ray dark-field imaging, grating-based phase-contrast imaging, cultural heritage

## Abstract

If ancient documents are too fragile to be opened, X-ray imaging can be used to recover the content non-destructively. As an extension to conventional attenuation imaging, dark-field imaging provides access to microscopic structural object information, which can be especially advantageous for materials with weak attenuation contrast, such as certain metal-free inks in paper. With cotton paper and different self-made inks based on authentic recipes, we produced test samples for attenuation and dark-field imaging at a metal-jet X-ray source. The resulting images show letters written in metal-free ink that were recovered via grating-based dark-field imaging. Without the need for synchrotron-like beam quality, these results set the ground for a mobile dark-field imaging setup that could be brought to a library for document scanning, avoiding long transport routes for valuable historic documents.

## 1. Introduction

Reading historical manuscripts can become impossible for various reasons, especially if the document has become so fragile over time that it cannot be opened. There are multiple approaches to recover such writings or even paintings, such as terahertz imaging [1,2], X-ray fluorescence, X-ray phase-contrast and standard X-ray imaging. X-ray phase-contrast was used by Mocella et al. [3] at the European Synchrotron Radiation Facility for a computed tomography of papyrus rolls that were carbonised during the eruption of Mount Vesuvius in 79 AD. In 2021, Michelin et al. [4] used X-ray fluorescence spectroscopy to distinguish different iron gall inks by determining traces of various metal elements contained in the inks. By this, a redacted part of a letter written by Marie-Antoinette during the French Revolution was recovered. It is even possible to image a whole book in a 3D X-ray CT scan at a synchrotron [5], or at a common laboratory source if it is written with metallic inks [6,7,8] or carved into bamboo scrolls [9]. Fully automated, virtual unrolling of the CT data makes the documents readable by the naked eye [10,11,12].

In this work, we present data of metal-free samples taken with a laboratory-based dark-field imaging setup instead of a synchrotron beamline. For this, we use a grating-based imaging setup powered by an X-ray source with a microscopic source spot size. Generally, there are different approaches to exploit X-ray phase-contrast [13,14,15,16,17,18]. At synchrotrons, a long propagation distance between the sample and the X-ray detector [19,20,21,22,23,24] or an imaging setup based on X-ray gratings [25,26,27,28] can be used for phase-contrast. Commercially available laboratory X-ray sources allow propagation-based or grating-based phase-contrast as well, if the requirement of a small spot size is met, providing sufficient spatial coherence [29,30,31,32]. For high-power laboratory X-ray sources with larger spot sizes and rotating anodes, grating-based phase-contrast can be implemented with a three-grating setup using an additional source-grating [33]. The differential phase-contrast probes the diffraction within a sample instead of the attenuation, which is useful for low-z materials where absorption is low. The dark-field indicates small-angle scattering within the sample, giving an insight to structural material properties on a sub-pixel scale. Owing to these capabilities, grating-based phase-contrast and dark-field imaging are of great interest in the fields of medical imaging [33,34,35,36,37,38,39,40,41] or non-destructive testing [42,43,44], which includes studies on paper material [45] or archaeological findings [46].

For this work, we apply such a grating-based imaging setup [27] and focus on the capability of the dark-field to image letters that are not written with ink containing metal components.

For our proof-of-concept measurements, we produced samples with authentic paper and two different commonly used inks to mimic historical documents. The metal component of the iron gall ink allows detection in the attenuation image. Less absorption is expected from the plant based metal-free thorn ink. The results demonstrate, that the dark-field, as compared to the attenuation, exhibits significantly improved contrast for thorn ink against the paper substrate.

## 2. Materials and Methods

### 2.1. Imaging Setup and Sample Preparation

**Figure 1:**
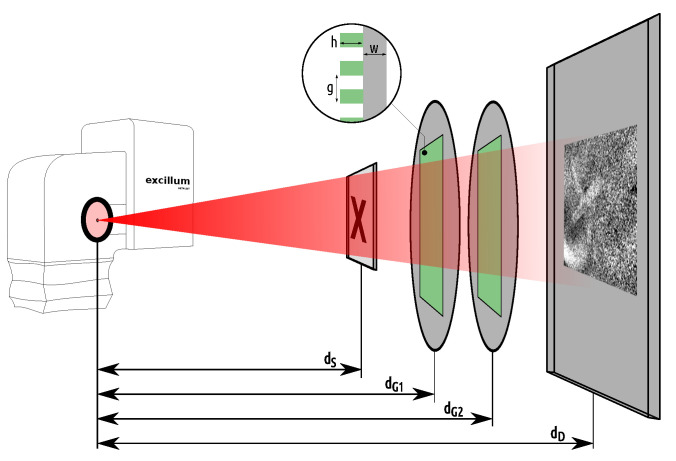
Sketch of the imaging setup showing the Excillum C2 metal-jet X-ray source head on the left, operated at a power of 58 W and 55 μm spot size. The cone beam is depicted by the red rectangle with an intensity gradient. The paper sample is situated at a distance of $d_{S}=90\;$cm from the source, followed by the phase grating G1 and the analyser grating G2 at the distances $d_{G1}=100\;$cm and $d_{G2}=120\;$cm, respectively. Placed at $d_{D}=138\;$cm is the X-ray detector with 49.5 μm pixel size. The zoom-in shows the grating structure with the grating pitch *g*, the grating bar height *h* and the wafer thickness *w*.

The setup for imaging the paper samples features a two-grating configuration and an Excillum C2 metal-jet X-ray source. The setup is sketched in Figure 1. It has a total length of $d_{D}=1.38\;$m from the source to the detector. The detector is a Teledyne DALSA Shad-o-Box 6k HS flat panel that has a CMOS sensor with a pixel size of 49.5 μm coupled directly to a Gadox scintillator. The sample is placed at $d_{S}=0.90\;$m from the source, yielding a geometric sample magnification of $M=d_{D}/d_{S}=1.53$ and an effective pixel size of 32.3 μm in the object plane. Located between the sample and the detector are the phase grating G1 at 1.00 m and the analyser grating G2 at 1.20 m. The grating periods are $g_{1}=10\;\mu$m and $g_{2}=6\;\mu$m, respectively. Both gratings are manufactured on 500 μm thick 4 inch wafers made of polyimide (G1) and graphite (G2). In contrast to standard silicon wafers, these low-absorbing wafers reduce beam-hardening to maintain a high flux for the soft X-ray spectrum. The gratings are optimised for a compact high-visibility setup at a design energy of 11 keV and were originally manufactured for use at a large-scale research facility [47,48]. The anode of the X-ray source is a jet of liquid-metal alloy consisting of indium and gallium. The source is operated at an acceleration voltage of 30 kV, so the spectrum shows strong line emission around the gallium Kα line at 9.3 keV, which is close enough to the design energy of the gratings.

Grating-based phase-contrast imaging without a source grating requires spatial coherent X-rays. This is achieved by a small source spot size [31,49]. For the used grating setup, the optimal spot size setting of the source was found at 55μm. With this, the source was operated at a current of 1.93 mA and an acceleration voltage of 30 kV, yielding a power of 58 W. Omitting the additional source grating simplifies the alignment procedure and improves the flux efficiency, because a source grating would absorb a significant part of the X-ray photons. The two gratings are mounted on a rigid, compact fast-alignment setup [50] for stable operation. The imaging setup is sensitive to movements of the source position [35]. Such a drift of the source position can occur if the X-ray head is not properly temperature stabilised in a range of $\pm 0.2{\;}^{\circ }$C. Despite the use of a correction algorithm [51], part of the source-drift artefacts can remain in the form of bent stripes spanning large parts of the image.

Both of the presented samples are manufactured from historically authentic materials. On the hand-crafted cotton paper with a density of 90 g/m${}^{2}$, one or multiple letters are written by hand with a quill and two different inks. We manufactured the inks according to authentic medieval recipes. The thorn ink is made from the bark of blackthorn [52]. The iron gall ink is made from oak gall and iron sulphate [53]. These inks are, besides the carbon ink, two of the three most widely used inks throughout the late antiquity and the medieval period [54]. The plant based thorn ink was historically used for its red-brown colour and because it is lightfast, water resistant and causes no ink corrosion to the paper. One sample also features the letter *X* written without any ink. It is written with the backside of a ballpen, so that the image shows only the deformation of the paper due to the pressure typically applied during writing. Each sample consists of only one sheet of paper with the letters written on it. A written document in an envelope or several overlapping pages, for example, pose an additional challenge, since every further layer of paper adds more background to the X-ray images.

### 2.2. Image Acquisition

The image acquisition is based on the phase-stepping technique [27]. This means that the analyser grating G2 is stepwise translated orthogonal to the grating bars while recording one detector frame for each step. These are the so-called phase-steps. In each detector pixel, this results in an intensity variation I(s) that can be described reasonably well by a sine function with [27]

(1) $$\begin{array}{c} I\left(s\right)=\hat{I}+A\cdot \sin\left(\frac{2\pi s}{p}\;+\phi \right)\;, \end{array}$$

where *s* is the phase-step number, $\hat{I}$ is the intensity offset, *A* is the amplitude of the sinusoidal variation, ϕ is the phase offset and p=d/g is the relation between the width of one phase-step *d* and the grating pitch *g*. The parameters are extracted from the data for every detector pixel by fitting Equation (1) to the corresponding stepping curve. However, since the source-drift causes a slowly progressing shift of the phase-step positions, a correction algorithm is applied that estimates the source position from the whole detector images [51]. While this significantly improves the fits, part of the source-drift artefacts can remain in the resulting images. The attenuation Γ, the dark-field Σ and the differential phase-contrast *D* are defined as [27,55].

$$\begin{array}{c} \Gamma =-\ln\left(\frac{{\hat{I}}_{\mathrm{obj}}}{{\hat{I}}_{\mathrm{ref}}}\right),\;\;\;\;\;\Sigma =-\ln\left(\frac{V_{\mathrm{obj}}}{V_{\mathrm{ref}}}\right)\;=-\ln\left(\frac{A_{\mathrm{obj}}/{\hat{I}}_{\mathrm{obj}}}{A_{\mathrm{ref}}/{\hat{I}}_{\mathrm{ref}}}\right),\;\;\;\;\;D=\phi_{\mathrm{obj}}-\phi_{\mathrm{ref}}\;. \end{array}$$

Here, obj and ref mark the parameters for the object and reference steppings, because the procedure requires a reference that is taken with the same imaging setup, but without an object in the beam path. The visibility, defined as $V=A/\hat{I}$, is also a quality measure for the grating interferometer, since a higher visibility improves the dark-field image quality [56]. For the presented setup, we reach an average visibility of 53% in the region of interest in the reference images. The dark-field and the attenuation are unit-less, since they are relative quantities normalised by the reference image.

A total of 30 phase-steps were used for each object and for each reference measurement, spread equally over two periods of the grating G2. The acquisition time per phase-step was 10 s, amounting to a total illumination time of 300 s, hence a total of 579 mAs. Facing this level of radiation, the paper is not expected to take any long-term damage [57]. The obtained attenuation and dark-field images are filtered by a 3 × 3 median filter for a reduction of image noise. The attenuation image corresponds to the standard radiography, while the dark-field highlights small-angle scattering in the sample [58]. The scattering diminishes the coherence of the X-rays, thereby decreasing the visibility of the stepping-curve, causing higher dark-field values in the resulting image.

### 2.3. Data Analysis

To compare the image contrast of the letters against the paper, the dark-field and the attenuation images are first separated into a region of interest (ROI) and a background region. For this, the noise in the respective image is first reduced by use of a sliding-window filter. Then, a threshold is set, separating most of the ROI from the background. The next step is to intervene by hand, if larger parts of the image are allocated to the wrong region. Finally, small fragments are removed and the remaining ROI is slightly enlarged by dilation and erosion, to form slightly larger, smoother clusters. For clarity, the resulting ROI is always provided in an extra image within the respective figure.

To analyse the image quality of the recovered letters, the pixel values of the separated image regions can be plotted in histograms. The histograms are normalised for better comparability, since the number of pixels $N_{\mathrm{ROI}}$ in the ROI is usually smaller than the pixel number in the background region $N_{\mathrm{BG}}$. For a combined illustration of the separation in the dark-field and attenuation images, we produced scatter plots. There, the dark-field values are plotted versus the corresponding attenuation values for each pixel of the image with a colour code for the ROI and the background. The ROI data is displaced relative to the background data in either the horizontal if there is attenuation contrast, or in the vertical direction if there is dark-field contrast between the letters and the paper, or it is displaced by a superposition of both.

For quantification, we use the mean pixel value $M_{\mathrm{ROI}}$ within the ROI and in the background $M_{\mathrm{BG}}$ to define the contrast-to-noise ratio (CNR) as

(2) $$\begin{array}{c} \mathrm{CNR}=\frac{S}{\sigma }\;=\;\;\frac{M_{\mathrm{ROI}}-M_{\mathrm{BG}}}{\sigma }\;, \end{array}$$

where $S=M_{\mathrm{ROI}}-M_{\mathrm{BG}}$ is called the distance of the mean value of the ROI from the background mean value, while σ is the standard deviation of the background pixel values. Here, we approximate $\sigma =\sigma_{\mathrm{ROI}}=\sigma_{\mathrm{BG}}$ and use only the standard deviation of the background, since it is representative for the pixel value distribution in the ROI. The CNR is calculated for both the dark-field and the attenuation. It compares the signal distance (*S*) to the level of noise in the image. For the detection of whole letters, it is reasonable to also regard the size of the letter, or the pixel number of its region. This motivates one to extend Equation (2) to define the detectability of a region as

(3) $$\begin{array}{c} R=\;\frac{1}{\sqrt{\frac{1}{N_{\mathrm{ROI}}}+\frac{1}{N_{\mathrm{BG}}}}}\;\cdot \mathrm{CNR}\;. \end{array}$$

Here, instead of the standard deviation σ, the standard error $\sigma_{N}=\sigma /\sqrt{N}$ is used for definition, which includes the pixel number *N* in the region of interest ($N_{\mathrm{ROI}}$) and in the background ($N_{\mathrm{BG}}$). However, even when ink is detectable, single letters can still be unreadable since the gaps between the letters require the same area to be detected. Otherwise, fine features of the letters get lost, or letters which are close to each other merge into one large ink stain. Furthermore, since the thorn ink is deposited on the paper by hand with a quill, the amount of ink varies throughout the sample which influences the signal in the images.

## 3. Results

### 3.1. Thorn Ink Sample

**Figure 2:**
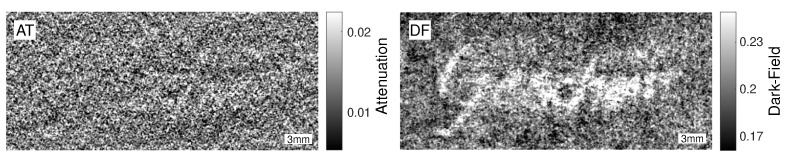
Attenuation (**left**) and dark-field image (**right**) of the word *Gescheit* written with thorn ink on 90 g/m${}^{2}$ cotton paper. The scale bar is 3 mm wide in the object plane.

**Figure 3:**
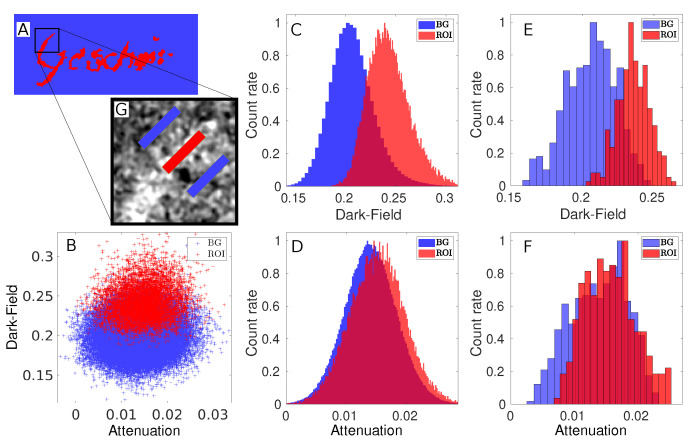
Quantitative evaluation for the thorn ink sample. (**A**) Separation mask of the region of interest (ROI) marked in red and the background region marked in blue. (**B**) Scatter plot of the dark-field values versus the attenuation for the ROI (red) and the background (blue). (**C**) Histogram of the ROI dark-field values (red) and the background (blue), showing a separation of the two peaks. (**D**) Histogram of the attenuation values from the same regions in the same colour code. (**E**) Dark-field histogram for the two blue background markers and the red ROI marker on the letter *G*, as can be seen in the zoom-in. (**F**) Attenuation histogram for the three markers. (**G**) Zoom-in showing the red marker on and two blue markers next to the letter *G*.

The attenuation and the dark-field image of the thorn ink sample are presented in Figure 2. It is the only sample in this work consisting of a full word. The German word *Gescheit* is mostly readable in the dark-field, but in the attenuation it is more difficult to make a clear statement. The word faintly shows up in the attenuation, especially for the letters *G* and *h*. However, without prior knowledge, the word would not be readable. To evaluate the difference between the two images, the sample area was separated. This can be seen in Figure 3A, where the region-of-interest (ROI) and the background region are marked in colour code. Due to the fine features of the letters and image noise, the regions have a rather rugged border instead of a smooth line of separation. A certain level of cross-talk between the regions is therefore expected but can be tolerated, since the same separation mask is used for both the attenuation and the dark-field images. Thus, the comparison of the two images is still valid for this specific sample with this specific ROI. A non-contaminated separation that covers only a small part of the sample is regarded later in this section.

The histogram that shows the separated dark-field image is given in Figure 3C. The distance between the ROI and the background values is S =0.0414. From this and the background standard deviation σ=0.0190, the contrast-to-noise ratio calculates to CNR =2.18. With the background pixel number $N_{\mathrm{BG}}=384,349$ and the pixel number $N_{\mathrm{ROI}}=32,751$ in the ROI, the detectability calculates to R =379. These results match the appearance of the histogram. There, the two peaks are clearly displaced to each other, although the distributions still partly overlap. For the same regions in the attenuation image, the histogram is given in Figure 3D. Here, the two distributions overlap almost completely. With a distance between the means of S =0.0012 and a background standard deviation of σ=0.0043, the contrast-to-noise ratio for the attenuation lies at CNR =0.29, which is more than seven times smaller than the dark-field CNR. The detectability for the attenuation lies at R =50.3. The scatter plot in Figure 3B combines the information from both histograms. The ROI data in the scatter plot is clearly displaced on the dark-field axis towards smaller values. The small displacement of the attenuation is hardly visible. Thus, the plot shows in one look that for thorn ink the image contrast in the dark-field is more pronounced than in the attenuation.

As a next step, we wanted to investigate the detectability of fine ink features. Therefore, a clean separation of a small part of the sample is defined by three markers (regions), shown in the zoom-in of Figure 3G. The central marker is located on the upper tip of the letter *G*, so that it only contains signal stemming from the thorn ink. Two background markers on the left and right side of the letter are placed with equal distance to the first marker. It is reasonable to place two background markers symmetrically, since the background varies throughout the image. The histogram in Figure 3F shows the distribution of the combined two background markers and the ink marker in the attenuation image, while Figure 3E shows the corresponding histogram for the dark-field. For the latter, the distance between the mean values for the pure cotton paper and the ink-covered part of the sample can be observed directly. With σ=0.0172, the distance $S_{\mathrm{DF}}=0.0304$ and a number of $N_{\mathrm{ROI}}=266$ pixels within the ROI marker, the dark-field contrast-to-noise ratio yields CNR =1.76 and the detectability R =23.5. For the attenuation image, the evaluation of the same markers yields a ratio of CNR =0.44 and a detectability of R =5.85. Both are around four times smaller in the attenuation than in the dark-field.

Based on these values, we define the amount of pixels in an area that is required for detection of thorn ink in the sample. For this definition, we use Equation (3) and insert the distances of the means of the pixel values determined within the markers in the dark-field ($S_{\mathrm{DF}}$) and in the attenuation ($S_{A}$). We further set $N_{\mathrm{BG}}=2N_{\mathrm{ROI}}$ to account for the two background markers. To achieve a confidence level of five sigma, we set R =5. With this, Equation (3) yields a result for $N_{\mathrm{ROI}}$, giving the number of pixels that is equivalent to the smallest size of an ink covered area that can be detected. The defined criterion leads to a necessary number of pixels of $N_{\mathrm{ROI}}=12$ for the dark-field. For this example, detection is thus possible for an ink-covered area of 0.013 mm${}^{2}$ or a square of 0.11 mm edge length. Using the same definition for the attenuation data, the required number of pixels calculates to $N_{\mathrm{ROI}}=182$, or an area of 0.19 mm${}^{2}$ which translates to a square of 0.44 mm edge length. This edge length is close to the line width of the letter *G* in the dark-field, which lies between 0.48 mm and 0.58 mm. Looking at the attenuation image of the full sample again, this result appears reasonable. With prior knowledge, it almost seems possible to see the writing, especially the letters *G* and *h*. In comparison however, the dark-field shows the ability to detect four times finer lines of thorn ink and produces a more than seven times higher CNR for the full image of the thorn ink sample.

### 3.2. Combined Iron Gall and Ink-Free Sample

**Figure 4:**
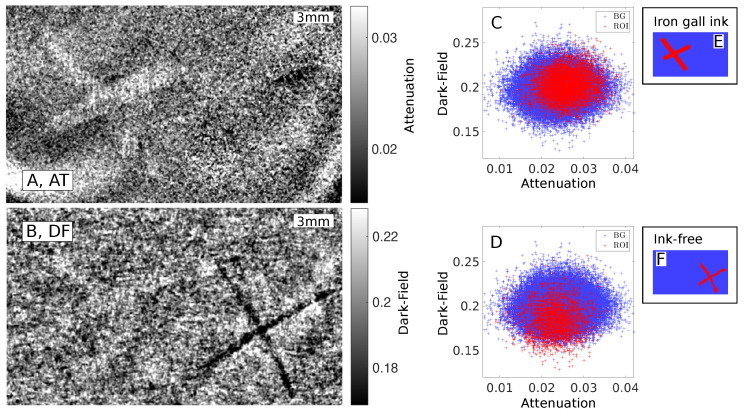
Attenuation (**A**) and dark-field (**B**) image of the sample that features an *X* written in iron gall ink (on the left side) and a further *X* written without ink (right side). The scatter plots show the distribution of the attenuation over dark-field values in the ROI (red) and the background (blue). The scatter plot in (**C**) uses the separation mask for the iron gall ink, depicted in (**E**), while the plot in (**D**) uses the separation mask for the ink-free *X*, shown in (**F**).

In this section, a comparison between two differently written letters is made on the basis of a sample that features the letter *X* written once in iron gall ink and once without any ink. The attenuation image (Figure 4A) shows the *X* written in iron gall ink clearly readable, overlaid by some source-drift artefacts, that could not be completely removed during the image processing of the phase-stepping data. The second *X*, written without ink, is not visible in the attenuation image. In the dark-field (Figure 4B), the correction algorithm was able to better suppress the source-drift artefacts. The iron gall ink letter is hardly visible, but the ink-free *X* becomes clearly visible. The scatter plot in Figure 4C shows the data separated for the iron gall ink letter by the mask shown in Figure 4E. The scatter plot shows a clear displacement on the attenuation axis for the iron gall ink, whereas the displacement in the direction of the dark-field axis is clearly smaller. This is in agreement with the contrast-to-noise ratios calculated for this configuration. In the attenuation image, the iron gall ink exhibits a ratio of CNR =0.48 and a detectability of R =76.1. In the dark-field, the iron gall ink exhibits a ratio of CNR =0.30 and a detectability of R =47.0. This also matches the appearance of the images, since the iron gall ink is visible more clearly in the attenuation image than in the dark-field. No beam-hardening correction is applied to the data, so this effect could be contributing to the dark-field signal of the *X* written with iron gall ink.

For the ink-free letter, the situation is different. In the attenuation image, the letter cannot be observed, but there are some bent stripes in the region, which are source-drift artefacts. The dark-field image (Figure 4B) clearly shows the X-shape of the letter. The scatter plot for the region of the ink-free *X* in Figure 4D shows a clear differentiation for the dark-field. This fits to the ratio for the dark-field of CNR =0.97 and the detectability of R =106. For the same ROI (see Figure 4F) in the attenuation, the ratio amounts to CNR =0.15 and the detectability is R =16.1. This CNR is less than a fifth of the dark-field CNR of the letter. Among all four combinations of this sample, it is the lowest CNR and also the lowest detectability. Interestingly, the CNR and detectability are still relatively high, but this is most likely due to the source-drift artefacts. Thus, it is fitting that the ink-free *X* cannot be observed in the attenuation. This confirms the clear advantage of dark-field imaging for the ink-free sample.

The letter *X* of the ink-free sample in the dark-field image appears darker than the background, which indicates lower dark-field values. This corresponds to less reduction of the visibility, so it appears that the deformed paper causes less small-angle scattering than the surrounding paper. This may be due to the force of the ballpen causing the fibres in the paper to arrange more regularly, thereby effectively reducing the gaps between them. Opposed to that, the thorn ink appears brighter in the dark-field than the surrounding paper, meaning the thorn ink increases small-angle scattering in the sample. The reason for this will be the subject of future studies.

## 4. Conclusions and Outlook

The presented results demonstrate that dark-field imaging can be advantageous for the detection of thorn ink at a standard laboratory X-ray source. The result of the previous studies, in which metal-based ink is visible by attenuation contrast due to the strong X-ray absorption of the metal components, was confirmed for our imaging setup. However, without the absorbing grating setup in the beam path, the higher X-ray flux would allow to record the attenuation images with less illumination time. This would also avert the source-drift artefacts. For the presented dark-field and attenuation images, the contrast of the written letters was quantified by the contrast-to-noise ratio and the detectability, based on the images being separated into the region-of-interest and the background. The results were visualised in histograms and scatter plots, allowing a quick estimation of whether the dark-field or the attenuation yields better image contrast. By selecting three small regions on the thorn ink sample, we defined a criterion for a limit of detecting small ink fragments, to show the advantage of the dark-field for this widely used ink. Furthermore, we found that no ink is required to visualise a letter in the dark-field image, if is written, for example, with the backside of a pen or a stylus. It appears that the applied pressure alters the dark-field properties of the paper sufficiently for detection of a written letter. For this case, the ink composition becomes irrelevant and readability only depends on the writing technique.

For the presented proof-of-concept study, the samples were kept in the simplest case possible, which is a single sheet of paper. Adding an envelope increases the background and requires good contrast from the ink. The next step would be to image an actual historical document. The authors recommend a sample with rather thin paper, since thick paper, especially with a prevalent internal structure, could obscure the written letters. A further step would be a computed tomography of a whole scroll or even a book at a laboratory dark-field imaging setup. For this, a better understanding of the origin and formation of the dark-field contrast between the paper and the ink will be helpful, especially concerning the influence of the sample position relative to the grating setup or the fibre orientation within the sample on the resulting dark-field images. Another interesting future study would be to image the drying process of different ink components in the dark-field to better understand the formation of the dark-field signal. Moreover, it would be interesting to test different authentic inks, for example carbon ink, as well as different writing techniques, ink-free marginal annotations, or letterpress printing with dark-field imaging.

This work represents an important step for bringing the analysis of fragile historical documents away from the synchrotron and towards the standard laboratory X-ray sources. This is crucial, because synchrotron beamtime is expensive and sets hard requirements for access. Furthermore, the transport of the fragile documents to large-scale synchrotron facilities is a risk that can be avoided by bringing a dark-field imaging setup to the archive.

## Data Availability

Not applicable.
